# Small Intestinal Imaging

**DOI:** 10.1155/2013/623091

**Published:** 2013-12-12

**Authors:** Marcela Kopáčová, Andre Van Gossum, Chris J. J. Mulder, Antonín Vavrečka, Jan Bureš

**Affiliations:** ^1^2nd Department of Medicine, Faculty of Medicine at Hradec Kralove, Charles University in Praha, University Teaching Hospital, Sokolska 581, 500 05 Hradec Kralove, Czech Republic; ^2^Clinique des Maladies Intestinales et du Support Nutritionnel, Service de Gastroentérologie, Hôpital Erasme, Route de Lennik 808, 1070 Brussels, Belgium; ^3^Department of Gastroenterology, VU University Medical Center, Amsterdam, The Netherlands; ^4^Department of Gastroenterology, Slovak Medical University in Bratislava, University Teaching Hospital of St. Cyril and Methodius, Antolska 11, 851 07 Bratislava, Slovakia

The small intestine was inaccessible for endoscopy for a long time. It was too far from both mouth and anus and was beyond endoscopist's reach. The history of investigation of the small bowel is quite short. In 1999 Mosse and Swain still stated in their work: “Enteroscopy remains the procedure in the gastrointestinal tract that is most inaccessible to endoscopy, and technical limitations severely impair the ability to advance and examine the small bowel reliably or completely” (Gastrointestinal Endoscopy Clinics of North America 1999; 9: 145–161).

There were only a few methods for small intestinal imaging in the past, including push and sonde enteroscopy, and radiological small-bowel follow-through studies. For all these methods, the diagnostic accuracy (the ability to diagnose and exclude disease correctly) is poor. The very last possibility was intraoperative enteroscopy. This method has been accepted as the ultimate diagnostic and/or therapeutic procedure for the complete investigation of the small bowel. It makes it possible to immediately solve pathological findings by endoscopic or surgical means and perform histopathological examinations of biopsy and/or resected specimens. By visualizing the mucosa, intraoperative enteroscopy can provide information for more precise surgery, thereby limiting resection. The very first investigation was performed in 1976. Intraoperative enteroscopy is an invasive method. The most widely used surgical approach is a standard laparotomy, but laparoscopically assisted enteroscopy has also been reported. Nowadays, this is the method assigned only for multiple transmural small intestinal lesions, which are too small to be found by a surgeon himself/herself, and unsuitable for endoscopic therapy because of its transmural character.

A break came in 2000 with a development of new tools: capsule enteroscopy and double balloon enteroscopy. Capsule enteroscopy was invented by Swain (World Congress of Gastroenterology, Los Angeles, 1994) and initial experience in 4,000 patients was published by Fritscher-Ravens and Swain in 2002 (Digestive Diseases 2002; 20: 127–133). Capsule enteroscopy allows for the end-to-end visualisation of the small bowel. However, the presence of a motility disorder or stricture may preclude successful investigation. Double balloon enteroscopy (and other deep enteroscopy methods: single balloon enteroscopy and spiral enteroscopy) is a neoteric technique, first published and introduced into clinical practice in 2001 by Yamamoto, inventor of this outstanding method. Double balloon enteroscopy allows complete visualisation, biopsy, and treatment in the small bowel. It is a gold standard for both investigation and therapy of small intestinal disorders recently. Confocal laser endomicroscopy is rather complementary and experimental method.

In radiological imaging the MRI is the most important method for the small intestinal investigation. The standard small bowel enema may not show early mucosal disease. CT and MRI enterography are gaining in usage; particularly as oral techniques have become more successful, obviating the problem of intubation. They both show bowel vascularity and mural and transmural changes. The role of abdominal ultrasound in experienced hands is also significant.

We bring nine articles in this special issue stressed on most interesting topics in diagnosis and treatment of small intestinal diseases and on rare and/or important small bowel illness.

New aspects of investigation of the small bowel are discussed in four articles: “*Transabdominal ultrasonography of the small bowel*,” “*Radionuclide small intestine imaging*,” “*Novel imaging enhancements in capsule endoscopy*,” and “*Utility of computed tomographic enteroclysis/enterography for the assessment of mucosal healing in Crohn's disease*.”

Refractory celiac disease are common and difficult topics in our everyday practice; that is why we chose one paper covering this topic to our issue “*Update on the diagnosis and management of refractory coeliac disease*”.

Small intestinal tumours, Cronkhite-Canada syndrome, cryptogenic multifocal ulcerous stenosing enteritis, and Whipple's disease are quite rare diseases, but it is necessary to know about them and give a thought to them (articles “*Small intestinal tumours*;” “*Cronkhite-Canada syndrome: review of the literature*;” “*Cryptogenic multifocal ulcerous stenosing enteritis: review of the literature*;” and “*Whipple's disease: our own experience and review of the literature*”).

We believe that selected manuscripts will help our readers to manage with the everyday practice and help them to understand to small bowel problems.

*Marcela Kopáčová* *Marcela Kopáčová* *Andre Van Gossum* *Andre Van Gossum* *Chris J. J. Mulder* *Chris J. J. Mulder* *Antonín Vavrečka* *Antonín Vavrečka* *Jan Bureš* *Jan Bureš*

